# Keap1-Nrf2 pathway: a key mechanism in the occurrence and development of cancer

**DOI:** 10.3389/fonc.2024.1381467

**Published:** 2024-04-03

**Authors:** Feilong Chen, Mei Xiao, Shaofan Hu, Meng Wang

**Affiliations:** ^1^ Sports Medicine Key Laboratory of Sichuan Province, Expert Centre of Sichuan Province, Institute of Sports Medicine and Health, Chengdu Sport University, Chengdu, China; ^2^ College of Bioengineering, Chongqing University, Chongqing, China; ^3^ Department of Biochemistry and Molecular Biology, Third Military Medical University (Army Medical University), Chongqing, China

**Keywords:** Keap1, Nrf2, cancer, structure, function, transcriptional regulation, prevention, clinical

## Abstract

The Keap1-Nrf2 signaling pathway is a major regulator of the cytoprotective response, participating in endogenous and exogenous stress caused by ROS (reactive oxygen species). Nrf2 is the core of this pathway. We summarized the literature on Keap1-Nrf2 signaling pathway and summarized the following three aspects: structure, function pathway, and cancer and clinical application status. This signaling pathway is similar to a double-edged sword: on the one hand, Nrf2 activity can protect cells from oxidative and electrophilic stress; on the other hand, increasing Nrf2 activity can enhance the survival and proliferation of cancer cells. Notably, oxidative stress is also considered a marker of cancer in humans. Keap1-Nrf2 signaling pathway, as a typical antioxidant stress pathway, is abnormal in a variety of human malignant tumor diseases (such as lung cancer, liver cancer, and thyroid cancer). In recent years, research on the Keap1-Nrf2 signaling pathway has become increasingly in-depth and detailed. Therefore, it is of great significance for cancer prevention and treatment to explore the molecular mechanism of the occurrence and development of this pathway.

## 1 Introduction

NRF2 (nuclear factor erythroid 2-related factor 2) is tightly regulated by different mechanisms at transcriptional, epigenetic, or ARE‐binding level; however, its key regulation is interceded by proteasome degradation mainly mediated by the repressor protein Kelch‐like ECH‐associated protein 1 (Keap1) (1). There are differences in the number of cysteine residues between human- and mouse-derived Keap1. There are 25 cysteine residues in mice and 27 in human-derived Keap1. Most can be modified by different oxidants and electrophilic reagents *in vitro (* 2). Nrf2 is an important redox-sensitive transcription factor that is conducive to improving the oxidative stress state of the body, promoting cell survival, and maintaining the redox homeostasis of cells by inducing and regulating the constitutive and inducible expression of phase II detoxification enzymes and antioxidant enzymes in cells (3–5). The Keap1-Nrf2 signaling pathway is a major regulator of the cell protection response, which is involved in endogenous and exogenous stress caused by reactive oxygen species (ROS) (6). This pathway is regulated with Nrf2 as the core. First, Nrf2 and small Maf protein bind to an ARE (antioxidant response element) in the regulatory region of the target gene. Second, Nrf2 binds with Keap1 to promote its degradation through the ubiquitin proteasome pathway (7). At the same time, this pathway also acts as a double-edged sword: Nrf2 activity protects cells against oxidative and electrophilic stress, while increasing Nrf2 activity contributes to cancer cell survival and proliferation.

In view of these remarkable findings, research on the Keap1-Nrf2 signaling pathway has become increasingly in-depth and detailed in recent years. Therefore, it is of great significance to constantly explore the molecular mechanism of the occurrence and development of this pathway for cancer prevention and treatment. In this review, the key molecular mechanisms of the Keap1-Nrf2 signaling pathway in carcinogenesis and development are summarized.

## 2 Structure and function of Keap1

### 2.1 Structure of Keap1

Keap1 was found and reported in 1999, and it belongs to the BTB Kelch protein family (8, 9). Keap1 is a negative regulator of Nrf2, which mainly binds with it in the cytoplasm to form a homodimer. Under normal circumstances, Keap1 interacts with Cullin3 (Cul3) and Rbx1 to ubiquitinate the Nrf2 protein and induce the proteasome to degrade it, thus preventing Nrf2 from being translocated to the nucleus and binding to the ARE site in DNA (10). The protein molecular weight of Keap1 is 69 kDa, which is located at position 19q13.2 of the human chromosome. Under normal circumstances, it is anchored to the actin skeleton in the cytoplasm.

**Figure 1:**
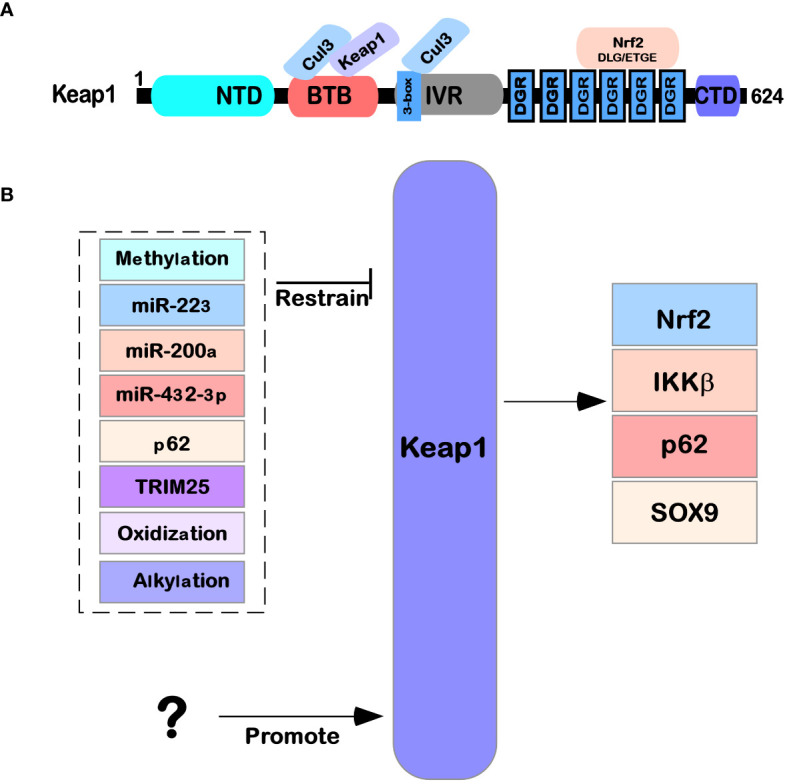
Structure and function of Keap1. **(A)** The protein structure of Keap1 and the functions of its domains. **(B)** The regulatory network of Keap1. Several microRNAs (miR-223, miR-200a, and miR-432-39) also affect Keap1 translation levels. The Keap1 protein is also regulated by various modifications (such as methylation, oxidation, glycosylation, and alkylation) after translation. At the same time, it is also affected by other factors (such as P62 and TRIM25) that regulate its expression.

Keap1 is mainly composed of the following five domains (**Figure 1A**): NTR domain (1–49 amino acid residues), BTB domain (50–179 amino acid residues) that can interact with Cul3, IVR domain (180–314 amino acid residues), six repetitive DGR domains (315–598 amino acid residues), and the final CTR domain (599–624 amino acid residues) (11, 12). The details are as follows: the BTB domain, also known as the POZ domain, has diverse functions and can participate in the polymer formation process of the Keap1 protein; for example, it can mediate the mutual recognition of Keap1 homodimers in the cytoplasm and bind to the E3 ubiquitination ligase complex dependent on Cul3 (13). At C151, a cysteine residue was found that is necessary for Keap1 to reduce E3 activity under electrophilic stimulation (14). The IVR domain (also known as the BACK domain) connects the BTB domain with the Kelch/DGR domain on the C-terminal side. Because this domain is rich in cysteine amino acid residues, it can regulate the activity of Keap1 protein. At the same time, Keap1 can interact with the 3-box double helix motif region near the N-terminal of Cul3 through the IVR domain (15). The Kelch/DGR domain of Keap1 is composed of six repetitive Kelch sequences, which contain tyrosine, tryptophan, diglycine, and other repetitive conserved amino acid residues; Keap1 has a homodimer that can interact with ETGE (high affinity) and DLG (low affinity) motifs in the Neh2 domain of the Nrf2 protein (16).

### 2.2 Expression of Keap1

By analyzing the RNA-seq data of 27 different human tissues (from NCBI), it was found that Keap1 was distributed in most human tissues and organs, such as the brain, adrenal gland, bone marrow, gallbladder, kidney, liver, spleen, and pancreas. Interestingly, the expression level of Keap1 RNA in different organs and tissues is quite different. For example, the expression level of Keap1 is high in the brain, kidney, and prostate but low in the bone marrow, pancreas, and salivary gland. The above data come from the National Center for Biotechnology Information (17).

### 2.3 Current status of Keap1 research

**Figure 2:**
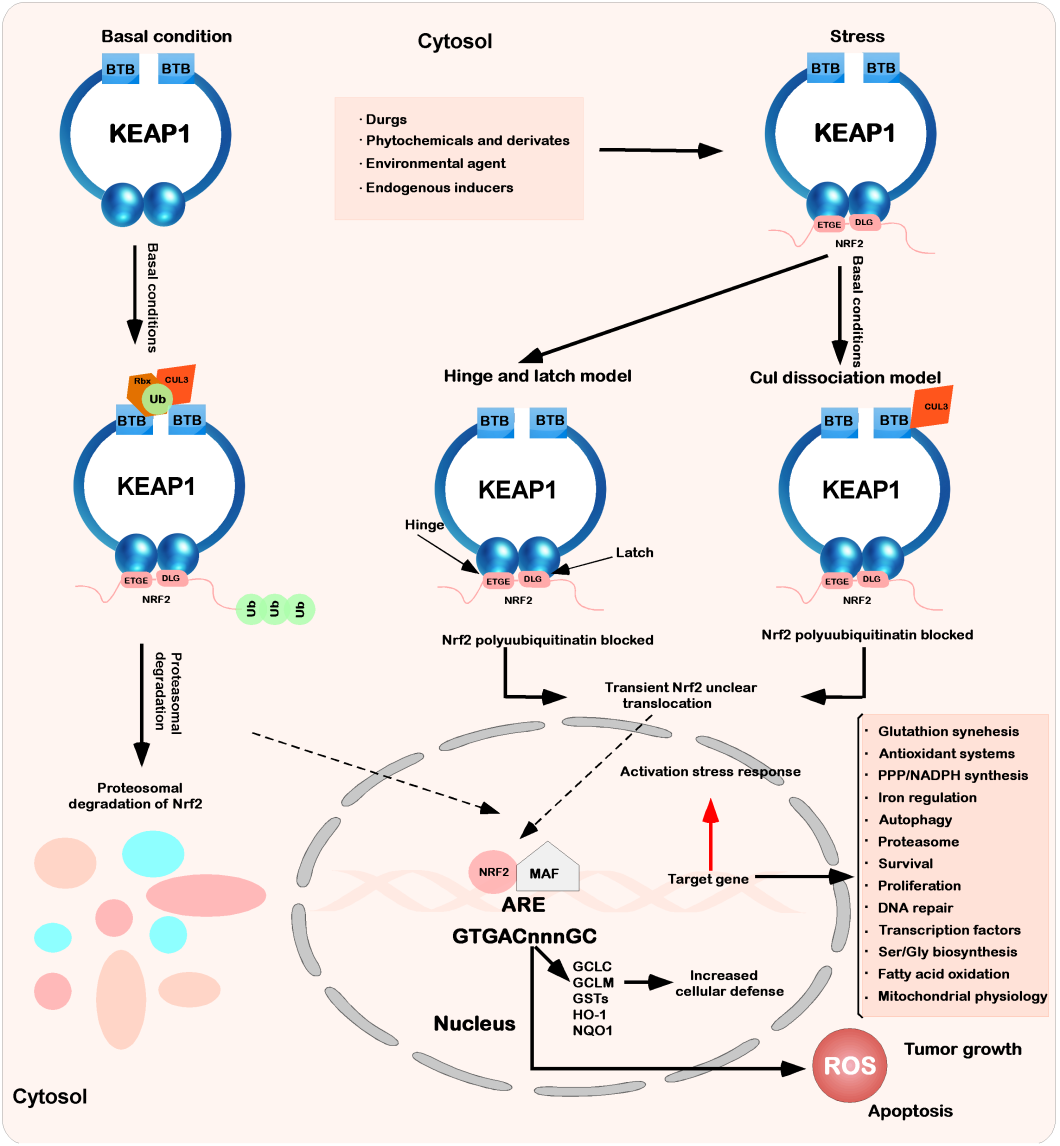
Interaction between Keap1 and Nrf2. Under basic conditions, Keap1 binds to Nrf2 through ETGE and DLEG, and Nrf2 is polyadenylated by the cul3-based E3 ligase complex. This polygeneralization leads to the rapid degradation of Nrf2 by the proteasome. At the same time, a small amount of Nrf2 escapes from the inhibition complex and reaggregates in the nucleus, mediating the expression of basic ARE-dependent genes and thus maintaining intracellular homeostasis. When stimulated by the outside world (drugs, phytochemicals and devivates, environmental agents, and endogenous inducers), the inducer modifies Keap1 cysteine and inhibits Nrf2 ubiquitination by dissociating the inhibition complex. According to the hinge and latch model, the modification of specific Keap1 cysteine residues leads to the conformational change of Keap1, leading to the separation of the Nrf2 DLG motif from Keap1. The ubiquitination of Nrf2 is destroyed, but binding to the ETGE motif still occurs. At the same time, in another model (Keap1-Cul3 dissociation model), the binding of Keap1 and Cul3 is destroyed under the action of electrophilic reagents, which leads to the escape of Nrf2 from the ubiquitination system. In these two models of Keap1-Nrf2, both will induce modification and inactivate Keap1, which will bind Nrf2. Therefore, the newly synthesized Nrf2 protein bypasses Keap1 and enters the nucleus, binds to the antioxidant response element (ARE), and drives the expression of the Nrf2 target genes GCLC, GCLM, NQO1, HO-1, and GST. At the same time, it will also affect other processes (glutathione synthesis, antioxidant systems, PPP/NADPH synthesis, iron regulation, etc.).

Keap1 is part of a ubiquitin ligase (cul3-rbx1 E3) complex that recruits pgam5, Nrf2, SLK, IKK β, P62, Sox9, Bcl-2 MIRO2, MAD2L1, and MYO9B, which are ubiquitinated and degraded and are involved in the regulation of multiple signaling pathways in cells (18) (**Figure 1B**). For example, in the Keap1-Nrf2 pathway in oxidative stress and metabolic processes, Nrf2 often causes corresponding case changes after aberrant expression occurs (19, 20). An increasing number of studies have also been used to demonstrate that Keap1 is a shuttling competent protein, i.e., shuttling back and forth in the nucleus and cytosol under specific conditions, whereas a nuclear export signal (leucine) is found in Keap1, and in large amounts of the nuclear protein prothymosin α that binds with Keap1, revealing that Keap1 is able to translocate from the cytoplasm into the nucleus (21). Later in the state of oxidative stress, Keap1 is also able to enter the nucleus and bind with Nrf2 again, allowing Nrf2 to translocate from the nucleus to the cytoplasm again and thus allowing Nrf2, under the mediation of Keap1, to be degraded by ubiquitination (22–25). Keap1 can act as an E3 ubiquitination ligase for p62 (a.k.a. SQSTM1), allowing p62 to be ubiquitinated for degradation and reducing cell death in disease (26) (**Figure 2**). Keap1 can also function as an IKK β, the role of E3 ligases that inhibit NF-κB expression of the pathway, which in turn inhibits cancer initiation (18).

It has been shown that Keap1 is not only a tumor suppressor but also a prooncogenic protein. In terms of disease initiation and progression, Keap1 has been implicated in several diseases, such as kidney disease, liver disease, inflammatory disease, sarcopenia, ophthalmic disease, neurodegenerative disease, cardiovascular disease, and ischemia/reperfusion injury. In some critical diseases (e.g., cancer), Keap1 has also been found to be somatically mutated, resulting in deregulation of its function in mediating ubiquitination, leading to cancer initiation and malignant progression. For example, somatic mutation of Keap1 in lung cancer, causing an increase in Nrf2 protein expression levels, leads to lung cancer initiation and progression. In addition, somatically acquired mutations have also been found in a variety of human cancer tissues, including head and neck (42%), ovarian (37%), gallbladder (30.7%), gastric (11.1%), colorectal (7.8%), clear renal cell (4.7%), liver (2.8%), prostate (1.3%), and glioma (1.7%), leading to the development of cancer (27–30). Keap1 mutations in the somatic fraction are shown in **Table 1**.

**Table 1:** Somatic mutations of Keap1 in various human cancers.

Cancer	Mutation
Lung cancer (18, 31, 32)	R71L, E117K, S144F, V155F, V167F, G186R, R204P, S224Y, L231V, S243C, P318-fs, P318L, R320Q, G333C, G364C, S404X, L413R, D422N, G423V, G430C, N469fs, N460fs, R470H, R470S, R479G, G480W, W497L, W544C, R554Q, R601W, G601W, G603W, E611D
Liver cancer (33–36)	N183S, N222K, D249Y, H274Y, R336Q, L342M, G464D, W554Q, R601W, G603W, E611D
Endometrial cancer (35)	C13T, T43M, R169C, H274Q, B320Q, Q337X, A356T, G367D, P384L, H424R, R507Q
The gallbladder adenocarcinoma (35)	P181-fs, G332-fs, S338L, G379D
Breast cancer (36)	C23Y, D256G, A522V
Adenocarcinoma of the appendix (36)	G558G
Gastric adenocarcinoma	Q82H, S233N, F280L, L281P, C288Y, G350S
Kidney cancer (35)	Y54D, M409T, W544R
Colorectal cancer (35)	S45P, I125V, T142M, D165N, A191D, M503I, R536H
Ovarian cancer (35, 37)	S45S, F107L, R116P, A159T, A188V, A189K, P412S, E611K
Esophageal cancer (35)	E138A, V324M
Pancreatic cancer (35)	V428V
Prostate cancer (35, 36, 38, 39)	M209L, Y255F, T314M, D357N, A407V
Malignant melanoma (35, 40, 41)	1518delC, 1519delG
Carcinoma of the urinary tract (35)	E218Q, E244K
Autonomic ganglion disease (35)	S351

Del is the abbreviation of deletion. Fs is the abbreviation of frame shift.

### 2.4 Regulation of Keap1

Several reports have indicated that the regulation of Keap1 mainly focuses on the transcription level, protein translation level (e.g., the regulation of miRNAs), and posttranslational modification clipping processing (e.g., oxidative modification, glycosylation modification, and alkylation modification), as shown in **Figure 1B**.

#### 2.4.1 Regulation of Keap1 at the transcriptional level

Keap1, at the transcriptional level, is directly regulated by methylated promoter regions (CpG islands). For example, in prostate cancers (39), non-small cell lung cancers (42), breast cancer (43), and colon cancers (44), where CpG islands act as Keap1 promoter regions, there is high methyl florescence and low expression.

#### 2.4.2 Regulation at the translational level

Some microRNAs (also known as microRNAs, miRNAs) have been reported to be regulated at the level of Keap1 protein translation. For example, transfection of miR-223 in HepG2 cells decreased the level of Keap1 protein expression, and transfection of its inhibitor significantly increased the level of Keap1 protein expression; the results showed that miR-223 was able to negatively regulate the protein expression of Keap1 in the cells (45). Another mic RNA, mir-200a, with low expression under fructose induction, activated the expression of Keap1, reduced the antioxidant capacity of the Keap1-Nrf2 pathway, enhanced cellular ROS, and activated the expression of lactamase (NLRP3), resulting in oxidative stress and lipid accumulation in cells, while the use of polydatin, which could effectively enhance the expression level of mir-200a, activated the antioxidant activity of the Keap1-Nrf2 pathway and could serve as one target site for the treatment of fructose-induced related disorders (e.g., liver injury and lipid deposition-like disorders) (46, 47). In addition, mir-432-3p has also been found to inhibit Keap1 expression in ESCC (esophageal squamous cell carcinoma), which in turn regulates the antioxidant activity of the Keap1-Nrf2 pathway (48–50).

#### 2.4.3 Regulation of Keap1 modification at the posttranslational level

##### 2.4.3.1 Oxidative modification

Under unstimulated conditions, redox reactions are in stable equilibrium in living cells; however, multiple stress responses are elicited in cells after redox stabilization is disrupted, and this condition is thought to be an important contributor to the development of numerous diseases. In regulating the balance between oxidation and reduction, EPS (Epalrestat), an electrophile, is used to activate relevant defenses against oxidative stress. For example, in the Keap1-Nrf2 are transcriptional pathway, which can be activated by CA (carnosic acid) and CS (carnosol) found in rosemary to rapidly synthesize endogenous antioxidant phase 2 enzymes. Notably, CA and CS are electrophilic only after oxidation and themselves belong to non-electrophilic species (51). In COPD (chronic obstructive pulmonary disease) patients, it was observed that Keap1 changed its conformation due to a long-term stimulation by free radicals or other chemicals in tobacco, leading to abnormal expression of the Keap1-Nrf2 pathway.

##### 2.4.3.2 Glycosylation modification

O-GlcNAc (O-linked N-acetylglucosamine) is a dynamic posttranslational modification (PTM) that reversibly modifies serine and threonine residues of thousands of nuclear, cytoplasmic, and mitochondrial proteins. It has been documented that the glycosylation (o-GlcNAcylation) modification of Keap1 at S104 is able to regulate the ubiquitination of Nrf2 and proteasomal destruction, and glycosylation at this site is not required for Keap1 to form a dimer. Meanwhile, o-GlcNAcylation at this site can also further optimize the conformation of Keap1 and promote ubiquitination of Keap1 substrates in a manner that enhances keap1-cul3 binding ability (50, 52).

##### 2.4.3.3 Alkylation modification

Alkylation of one or more of the 27 cysteine sulfhydryls of human Keap1 has been reported to result in ubiquitination or proteasome-mediated reduction of its substrates. However, alkylation of Keap1 can also occur in the presence of some electrophilic compounds (e.g., quinone methides, carbenium ions, epoxides, quinones, and quinoneimines, among others) (53). Notably, xanthohumol, as a natural compound, is also able to alkylate Keap1 (54, 55). Another endogenous metabolite is itaconate, which has anti-inflammatory metabolic functions and alkylates some of the Cys residues (e.g., C151, c257, c273, c288, and c297, of which C151 has the strongest electrophilic activity) on the Keap1 protein, enhancing the expression of its antioxidant and anti-inflammatory related downstream genes (56–59). The in-depth study of substances such as xanthohumol and itaconic acid may provide a new approach to the pathogenesis of related diseases caused by Keap1.

## 3 Structure and function of Nrf2

### 3.1 Structure of Nrf2

**Figure 3:**
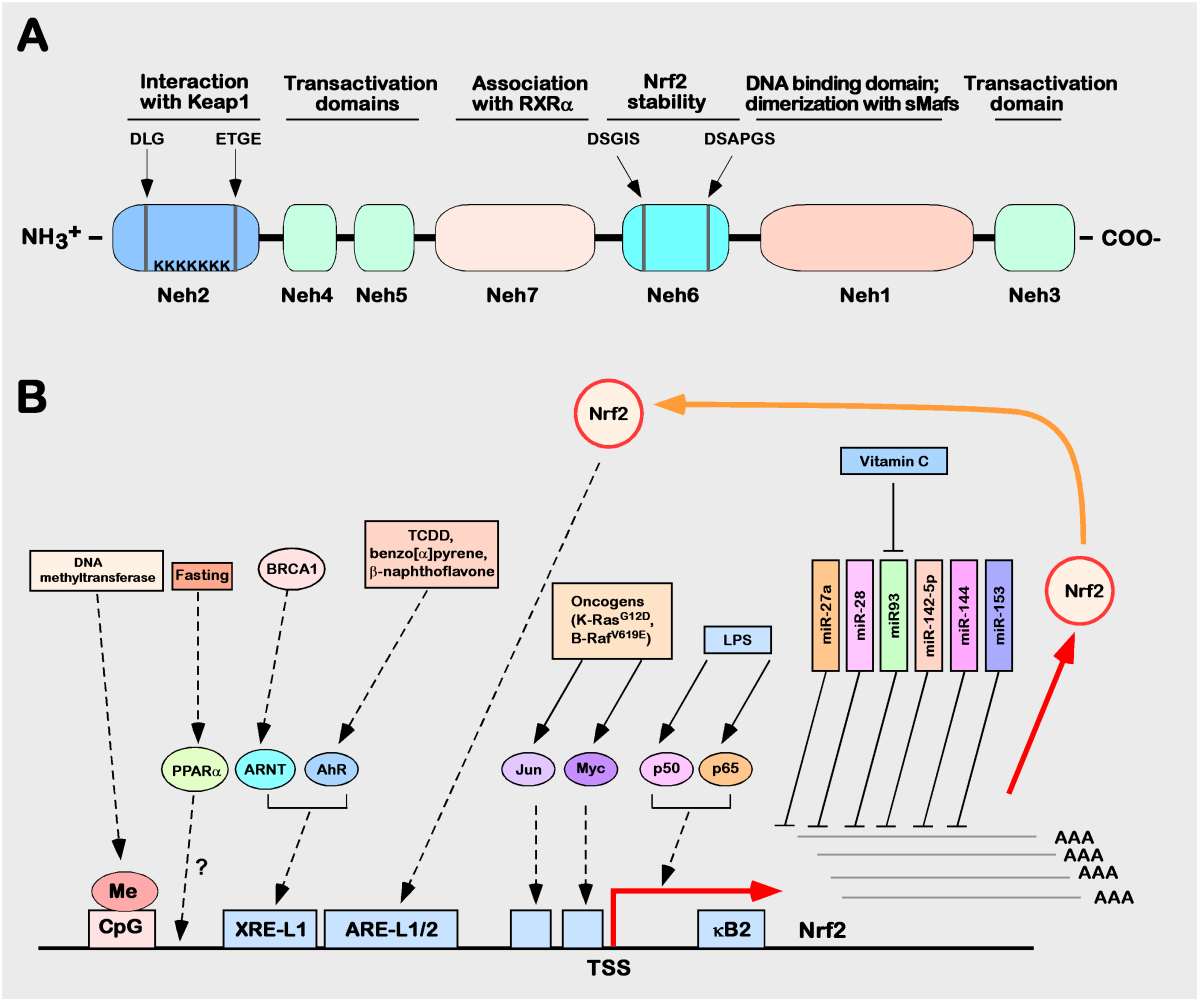
Structure and function of Nrf2. **(A)** Schematic diagram of the Nrf2 protein structure. **(B)** Schematic diagram of the regulation of human Nrf2 gene expression. The control mechanism of nuclear factor erythroid 2p45 related factor 2 (Nrf2) gene expression. The Nrf2 gene is depicted as the bottom of a solid black horizontal line graph, and the red right angle arrow represents the transcription start site (TSS). Breast cancer protein (BRCA) 1 increases the expression of Nrf2, which is mediated by ARNT. Lipopolysaccharide (LPS), as a factor promoting inflammation, can induce Nrf2 to recruit TSS (kB2) from the p50–p65 heterodimer through the nuclear factor (NF)-kB binding site. In the process of tumorigenesis and development, Nrf2 can be activated by many factors (such as Jun or Myc). Fasting increases the mRNA expression level of Nrf2, which may be mediated by peroxisome proliferator activation (PPAR). Many miRNAs, such as miR-27a, miR-28, miR-93, miR-142-5p, miR-144, and miR-153, inhibit the expression of Nrf2.

The Nrf2 transcription factor was first identified in 1994 in a human chronic myelogenous leukemia cell line by MOI et al. and was later determined to be located on human chromosome 2q31.2 (60). Nrf2, also named nfe2l2 (nf-e2-like 2), encodes a total of 605 amino acids and is composed of seven different domains, followed from the N-terminus to the C-terminus by Neh2, Neh4, Neh5, Neh7, Neh6, Neh1, and Neh3 (20, 61–63) (**Figure 3A**). The functions of each domain are specific and indispensable (64–66).

The Neh1 domain, which consists of the conserved CNC and bZIP domains, is critical for Nrf2 binding SMAF proteins in the nucleus to form dimers that recognize DNA sequences of target genes (67, 68). The Neh2 domain is located in the N-terminus of Nrf2 and contains two stretches of highly conserved amino acid sequences (29DLG31 sequence and 79ETGE82 sequence), which are able to bind with Nrf2’s inhibitor protein Keap1 to mediate Nrf2 degradation through the ubiquitination proteasome system (8, 9, 69). The C-terminal neh3 domain, neh4 domain, and neh5 domain are important domains for Nrf2 to exert transcriptional regulation of target gene activity (70–72). The neh6 domain is a serine-rich domain that contains two conserved amino acid motifs (343dsgis347 sequence and 382dsapgs387 sequence) that can be recognized by GSK-3 α/β-Trcp recognition, and deletion or mutation of either of these two sequences reduces β-Trcp-mediated ubiquitination, i.e., this domain is a critical negative regulatory domain that mediates proteasomal degradation of Nrf2 ubiquitination (73, 74). The neh7 domain was only formally defined in 2013 as a domain with a negative regulatory function through the retinal X receptor α (retinoic X receptor α, RXR α) interacting with this region and thereby repressing Nrf2 transcriptional activity (75).

### 3.2 Nrf2 expression

Analysis of Nrf2-related data (transcriptome and proteome data) in the HPA (Human Protein Atlas) database revealed its distribution in most human tissues and organs. Nrf2 expression has been detected in various organs, such as the brain, lung, kidney, liver, male and female reproductive organs, lymphoid tissue gallbladder, and muscle tissue, and in relevant cells, both in the cytoplasm and nucleus. It is worth noting that the expression levels of Nrf2 vary in different tissues or cells due to their functional and structural differences, while it is possible that there are some differences in the expression of protein and RNA levels in the same tissue or corresponding cells. In the brain, bone marrow, gastrointestinal, reproductive, and lymphoid tissues, the protein expression level of Nrf2 is relatively higher than that in other tissues, whereas the RNA expression level of Nrf2 is higher in esophageal tissues than in other tissues. The above data come from the National Center for Biotechnology Information (17).

### 3.3 Regulation of Nrf2

The related regulation of Nrf2 expression and activity can occur at the transcriptional level, mechanistically through miRNA-mediated regulation and through translational and posttranslational modifications. Because Nrf2 is a soluble protein, its regulation mainly occurs at the protein level, including protein−protein and posttranslational modification level regulation (76).

#### 3.3.1 Regulation of Nrf2 at the expression level (transcriptional level and miRNA-mediated mechanistic regulation)

Nrf2 can be activated or repressed at the transcriptional level through its own or other transcription factors; binding to ARE and XRE sites, such as ppar γ (peroxisome proliferator activated receptor γ) (77), MEF2D (myocyte enhancer factor 2D) (78), and AHR (aryl hydrocarbon receptor) (79), can directly activate Nrf2 expression, while p53 (79), p97 (80), and RXR α (81), among others, can negatively regulate Nrf2. Several miRNAs have been reported to regulate Nrf2 expression at the posttranscriptional level, such as mir27a, mir28, mir-93, mir-142-5p, mir-144, and mir-153. They are able to bind at the three-terminal noncoding region in the mRNA of Nrf2 to inhibit the expression of Nrf2 (76). Five CpG sequences are included in the promoter region of Nrf2, which, after hypermethylation modification occurs, can significantly inhibit Nrf2 expression (82). The mechanisms of the regulation of human Nrf2 gene expression are illustrated (**Figure 3B**).

#### 3.3.2 Regulation of protein translation and posttranslational modification by Nrf2

The activity regulation of Nrf2 protein can be regulated by several pathways; three pathways are cytoplasmic pathway regulation, endoplasmic reticulum pathway regulation, and nuclear pathway regulation. First, under normal circumstances, Keap1, in a homodimeric manner, recognizes DLG and ETGE sequences in the Neh2 domain of Nrf2 and anchors it in the cytoplasm; meanwhile, the N-terminus of the Keap1 protein recognizes and binds to Cul3, leading to rapid degradation by the proteasome after ubiquitination of Nrf2 (82, 83). There are also studies indicating that Nrf2 in the nucleus undergoes acetylation, leading to its binding to the alkaline region leucine zipper protein to antioxidant response elements, thereby triggering gene transcription (84). Second, in the regulation of ER stress, Nrf2 expression is suppressed through the transcriptional activation of the XBP1-HRD1 arm and the action of E3 ubiquitination-linked enzymes (85–87). Micro-RNAs (miRNA) can serve as a very powerful epigenetic regulator of Nrf2 (88). Third, Nrf2 is a major transcription factor that directly or indirectly significantly regulates over 2,000 genes. Although many of these genes are involved in maintaining redox balance, others are involved in maintaining balance between metabolic pathways that seem unrelated to oxidative stress (89). Nuclear regulation occurs through regulation by β-TrCP. It has been reported that in GSK3 β, following the sequential phosphorylation of dsgis (in the neh6 domain) of Nrf2, β-Trcp is activated; increasing the association of Nrf2 with the interaction force between β-TrCP accelerates the degradation of Nrf2 (73, 90, 91).

## 4 The Keap1–Nrf2 pathway as a therapeutic target

### 4.1 Effect of drugs on the expression of Keap1-Nrf2 pathway

Oxidative stress plays a key role in the pathogenesis of various human cancers (92, 93). Therefore, in some clinical studies, oxidative stress-related reactions have also been used to determine markers of human cancer (94). The Keap1-Nrf2 signaling pathway can prevent organ and cell damage caused by oxidative stress and protects against the occurrence and development of cancer (95). Because oxidative damage is common in carcinogenesis, the Keap1-Nrf2 signaling pathway is widely considered a potential therapeutic target for chemoprevention (93). The inducers of Nrf2 can play the role of chemopreventive agents in the following two ways: first, by preventing carcinogens from reaching their target sites, and second, by preventing carcinogens from interacting with important biological molecules (such as DNA and RNA) and proteins to play the role of chemopreventive agents (93). Although Nrf2 has chemopreventive potential in normal and precancerous tissues, it has also been shown to play a role in tumor cell growth and survival in malignant cells (7, 96). High levels of Nrf2 have been found in several types of human cancer cells. Mutations in Keap1 or Nrf2 lead to the constitutive expression of upregulated genes (97–99). The increased expression of Nrf2 can play a protective role in both normal and cancer cells. The increase in Nrf2 expression levels can lead to an increase in the expression of detoxification enzymes, cytoprotective proteins, and transporters. This allows cancer cells to gain advantages by enhancing cell proliferation and can cause drug resistance to chemotherapy (7, 65, 66, 96–100). Previous studies have shown that inhibiting Nrf2 in malignant cells can inhibit tumor growth and improve the efficacy of chemotherapy (100–102). After interfering with the PI3K/AKT and ERK pathways through natural flavonoids, Nrf2 was reduced at the mRNA and protein levels, making hepatoma cells sensitive to chemotherapy (103, 104). Interestingly, dimethyl fumarate, approved by the FDA as an Nrf2 activator, shows some anticancer activity. In fact, it seems to be an Nrf2 inhibitor at high concentrations (105). The p62 interacting with KEAP1 shows a good effect in HCC by downregulating Nrf2 activation (103).

### 4.2 Clinical application of the Keap1-Nrf2 pathway

The Keap1-Nrf2 signaling pathway is involved in both benign and malignant tumor diseases and may be used as a prognostic marker or therapeutic target. Nrf2 has also been shown to have an impact on the drug resistance of cancer (such as lung cancer, liver cancer, and thyroid cancer) to chemotherapy and radiotherapy (106–109). Therefore, in clinical trials, the expected effects of prevention and treatment can be achieved by targeting other components of the Keap1-Nrf2 pathway and its downstream signaling pathway. Clinical studies have shown that the mutation frequency of Keap1 and Nrf2 is approximately 25% in lung cancer patients. The prognosis of lung cancer patients with Keap1 or Nrf2 mutations is worse than that of lung cancer patients without this mutation (99). In addition, some studies have shown that the decrease in the expression level of Keap1 and the increase in the expression level of Nrf2 may also be related to poor prognosis. In general, Nrf2 is believed to contribute to both intrinsic and acquired resistance (110, 111). Nrf2-targeted genes involved in foreign biological metabolism can accelerate the metabolic inactivation of antitumor drugs; genes involved in drug transport can effectively reduce the intracellular drug concentration, and genes involved in thiosulfur synthesis can increase the drug tolerance of tumor cells. These multiple mechanisms together lead to chemotherapy resistance, which is one of the most important carcinogenic functions of Nrf2. In the process of thyroid cancer treatment, there are few alternative drugs (7). Proteasome inhibitors are a substitute for targeted anticancer drugs used in clinical thyroid cancer. Proteasome inhibition usually also leads to Nrf2 activation (112). The mechanism by which Nrf2 promotes thyroid cancer proteasome inhibitor resistance is not limited to the interaction with apoptosis regulators (ATF4, ORP150, etc.) but also includes direct regulation of cell redox status. Nrf2 not only promotes resistance to proteasome inhibitors but also promotes resistance to other experimental therapies (109, 113, 114). Nrf2 is upregulated in head and neck squamous cell carcinoma (HNSCC). Nrf2 reprograms a wide range of cancer metabolic pathways, and the most notable is the pentose phosphate pathway (PPP) (115). In cervical cancer, Nrf2 can activate EMT-related behaviors and promote cancer metastasis (116). There are also studies indicating that Nrf2 acts as a phenotypic stability factor in restricting complete EMT and plays an important role in coordinating collective cancer migration (117). In summary, a better understanding of the relationship between the activation of the Keap1-Nrf2 signaling pathway in cancer and the overall therapeutic effect, and the mode of interaction and the therapeutic relevance of this interaction, will help to further develop therapeutic drugs.

## 5 Discussion

**Figure 4:**
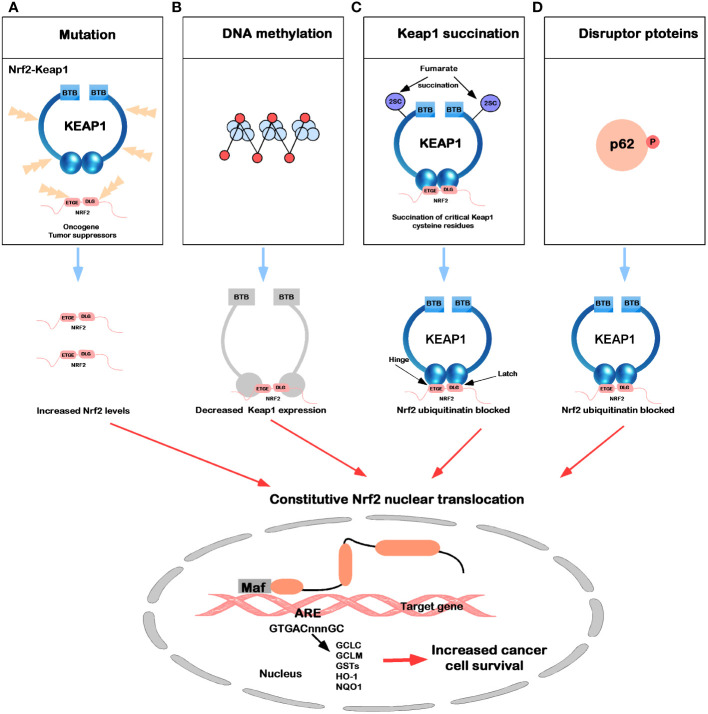
Mechanisms for constitutive nuclear accumulation of Nrf2 in cancer. **(A)** Somatic mutations in Nrf2 or Keap1 disrupt the interaction of these two proteins. **(B)** Hypermethylation of the Keap1 promoter in lung cancer and prostate cancer leads to decreased expression of Keap1 mRNA, thereby increasing nuclear accumulation of Nrf2. **(C)** In familial papillary renal carcinoma, the loss of fumarate hydratase activity leads to the accumulation of fumarate, which in turn leads to the succination of the Keap1 cysteine residue (2SC). **(D)** The accumulation of interfering proteins such as p62 and p21 can interfere with the binding of Nrf2 to Keap1, leading to an increase in nuclear Nrf2.

In the Keap1-Nrf2 signaling pathway, newly synthesized Nrf2 protein bypasses Keap1, translocates into the nucleus, and drives the expression of Nrf2 target genes, such as HO-1, NQO1, GCLC, GCLM, and GSTs (7). In **Figure 4**, five common molecular mechanisms of Nrf2 signaling activation in various cancers have been described (39, 118–123): (1) they are lost after Keap1 has been mutated in cells or Nrf2 has disrupted its binding domain with Keap1; (2) epigenetic silencing of Keap1 expression leads to defective repression of Nrf2; (3) accumulation of interfering proteins (such as p62) leads to dissociation of the Keap1-Nrf2 complex; (4) transcriptional induction of Nrf2 by oncogenic factors (e.g., K-Ras, B-Raf and c-myc); and (5) in familial papillary renal cancer, posttranslational modification of Keap1 cysteines by succinylation due to loss of fumarate hydratase activity deregulates the Keap1-Nrf2 signaling pathway.

The transcription factor Nrf2 has an important function in mediating cellular homeostasis (20, 76, 124, 125), playing a crucial role during tumor development (20, 125–129). It has been shown that tumor proliferation and Nrf1 are distinct and that malignant proliferation is inhibited after interfering with Nrf2 expression or knockdown (130–133). Upon inactivation of the tumor suppressor PTEN, the activity of the PI3K-Akt pathway increases, resulting in the elevated expression of Nrf2, which promotes cell proliferation (134); N-cadherin expression, a marker protein of EMT, was suppressed in cancer cells in which Nrf2 was inhibited or knocked down (135, 136), whereas E-cadherin expression was decreased in cancer cells in which Nrf2 was overexpressed (137). Currently, in most reports, Keap1 is a negative regulator of Nrf2, which is regulated through the ubiquitin proteasome system.

Accumulating evidence indicates that the Nrf2 signaling pathway is deregulated in many cancers, leading to aberrant expression of a Nrf2-dependent gene battery. Therefore, the development of therapies with anti-inflammatory activity mediated by Nrf2 is likely to have a major clinical impact. The ongoing Nrf2 signaling pathway is leading worldwide efforts to develop highly targeted therapeutic agents to control inflammatory symptoms and prevent and treat major diseases such as cancer and neurodegenerative diseases.

## Author contributions

FC: Conceptualization, Data curation, Formal analysis, Funding acquisition, Investigation, Methodology, Project administration, Resources, Software, Supervision, Validation, Visualization, Writing – original draft, Writing – review & editing. MX: Writing – review & editing, Data curation. SH: Data curation, Writing – review & editing. MW: Writing – review & editing, Data curation.
